# Hypertrophic lupus erythematosus hypertrophic lichen planus overlap responding to acitretin and anifrolumab

**DOI:** 10.1016/j.jdcr.2025.01.039

**Published:** 2025-03-04

**Authors:** Roxana A. Hojjatie, Brent Flickinger, Lauren N. Stuart, Misty D. Caudell

**Affiliations:** a Department of Dermatology, Emory University School of Medicine, Atlanta, Georgia; b Southeastern Rheumatology Alliance, Gainesville, Georgia; c Northeast Georgia Medical Center, Gainesville, Georgia; d Finan Templeton Dermatopathology Associates, Atlanta, Georgia; e Georgia Skin Cancer & Aesthetic Dermatology, Gainesville, Georgia

**Keywords:** acitretin, anifrolumab, chronic cutaneous lupus, cutaneous lupus, hypertrophic lichen planus, hypertrophic lupus, lichen planus, lichen planus-lupus erythematosus overlap syndrome, lupus

## Introduction

Cutaneous lupus erythematosus (CLE) and lichen planus (LP) are 2 autoimmune skin diseases with distinct pathophysiologies, clinical presentations, and management. CLE/LP overlap syndrome is a rare skin disease that contains clinical, histological, and immunological features of both diseases, which can delay diagnosis for several years.^1^ Because of the heterogenous presentation of CLE/LP overlap and the limited number of reported cases, consensus lacks on diagnosis and management. However, prior case reports have shown acitretin as a successful treatment option for CLE/LP overlap.^1^^,^^2^ Furthermore, the FDA recently approved the use of anifrolumab, a human monoclonal antibody binding type I interferon (IFN-1) receptor, for systemic lupus erythematosus. Because IFN-1 plays a role in the pathogenesis of CLE, the use of anifrolumab for CLE has been promising.^3^ We report an interesting case of a patient with CLE/LP overlap responding well to acitretin and anifrolumab.

## Case report

**Fig 1:**
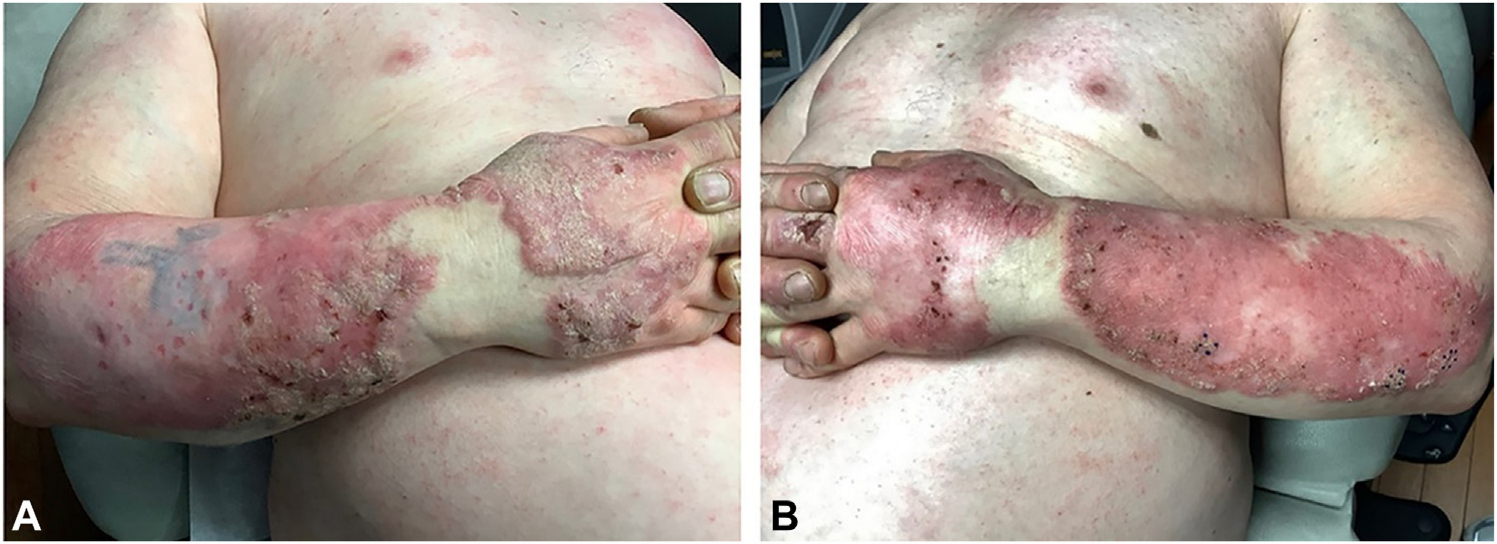
Hypertrophic lupus with lichen planus overlap in the right (**A**) and left (**B**) forearms in February 2020.

A 60-year-old Caucasian male with a longstanding history of discoid lupus erythematosus of the face, ears, and scalp diagnosed in 1988; hypertrophic CLE/LP overlap of the upper extremities diagnosed in 2006; and polyarthritis presented to the clinic with continued painful lesions of the upper extremities (Fig 1). His social history was notable for heavy sun exposure due to the patient’s occupation as an irrigation specialist (1984-2022). Additionally, he had a 44 pack-year history of smoking (1974-2018) with attempts at quitting throughout his treatment course. This patient had been on hydroxychloroquine 200 mg twice daily since 1998. The patient had previously failed several therapies prescribed by his rheumatologist or dermatologist for polyarthritis and chronic cutaneous lupus due to toxicity or lack of efficacy. These treatments included intralesional corticosteroids, methotrexate, mycophenolate mofetil, belimumab, rituximab, and abatacept. Additional medical history included multiple squamous cell carcinomas of the left forearm, hypohidrotic ectodermal dysplasia, Lynch syndrome, colon cancer, and hypertension. A biopsy of the patient’s right ventral distal forearm demonstrated verrucous epidermal acanthosis with hypergranulosis and hyperkeratosis and a band-like lymphocytic infiltrate abutting the dermal-epidermal junction, associated with dyskeratosis.

**Fig 2:**
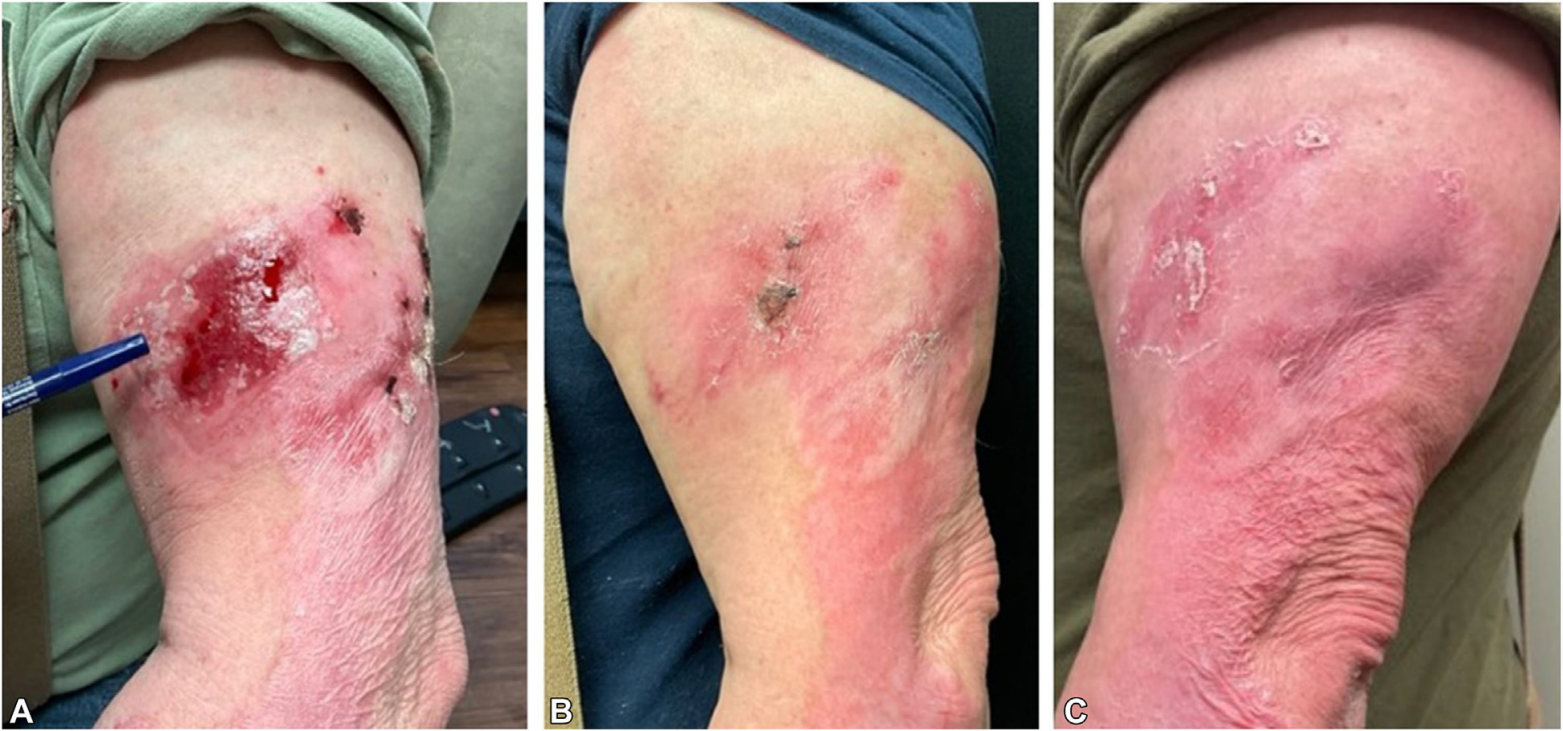
Before-and-after photos of the left upper extremity following acitretin, anifrolumab, and clobetasol 0.05% topical gel. **A,** May 2023. **B,** August 2023. **C,** June 2024.

**Fig 3:**
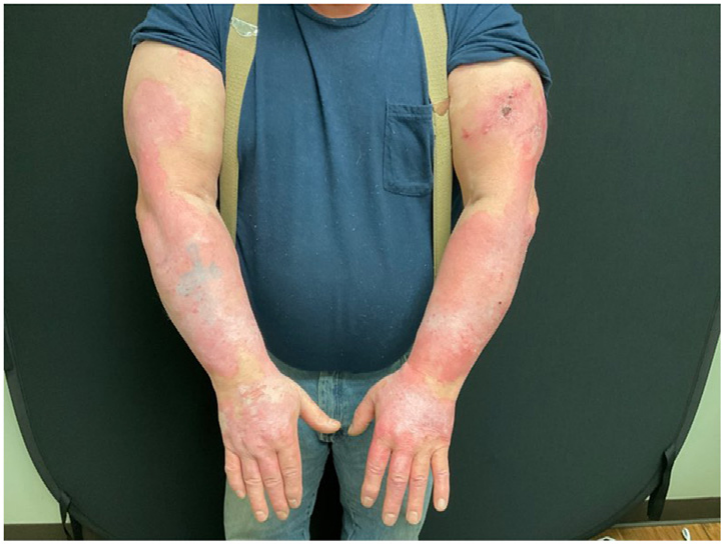
Dramatic disease improvement following acitretin and anifrolumab in May 2023.

On February 1, 2023, the patient was started on acitretin 25 mg every other day after counseling and review of his CBC, CMP, and lipid panel. After 2 months of treatment, the severity of his disease was unchanged. Acitretin was increased to 25 mg 5 days a week with a plan to increase daily, which the patient tolerated well. Topical steroids and antihistamines were also recommended for managing his hypertrophic LP. At his May follow-up, the patient’s hypertrophic CLE/LP had improved, though he continued to exhibit prominent granulation tissue on the left arm (Fig 2). Clobetasol 0.05% topical gel was prescribed for granulation. Coincidentally, at this appointment, the patient reported that he had recently started and received 2 infusions of anifrolumab-fnia (Saphenlo, AstraZeneca) by his rheumatologist for lupus management. In May 2023, photos of the patient’s upper extremities demonstrated remarkable and rapid improvement in the disease affecting his upper extremities (Fig 3).

Due to the coincidental similar timing of acitretin and anifrolumab, it was unclear which treatment was responsible for the dramatic improvement. In an effort to determine this and to minimize immunosuppression, anifrolumab infusions were discontinued in October 2023. However, this led to a flare of the patient’s CLE/LP, and anifrolumab was resumed. The patient has since been off acitretin for over 3 months with minimal signs of worsening disease, suggesting that continued improvement may be sustained with anifrolumab and topical steroids. The patient’s most recent treatment regimen includes anifrolumab and clobetasol 0.05% topical gel, and his skin lesions have improved dramatically.

## Discussion

CLE/LP overlap syndrome is an extremely rare cutaneous disease often affecting the extremities, face, and trunk. Clinical presentation often includes large, scaly, and painful plaques with resulting atrophy and a bluish-red color.^4^^,^^5^ However, patients may exhibit lesions that are consistent with either CLE or LP alone, making diagnosis challenging.^4^ Diagnosis relies on a combination of clinical, histological, and immunopathological findings of both diseases that are present concurrently.^4^

Increased interferons, particularly IFNs-1, play a key role in the pathogenesis of CLE.^6^ IFN-1 promotes leukocyte recruitment to the skin by triggering inflammatory cytokines, chemokines, and adhesion molecules and is generated in response to UV light, nuclear antigens, and immune complexes.^7^ Studies have also shown a role of IFNs-1 and IFN-gamma in cutaneous LP.^8^ Anifrolumab, a monoclonal antibody that selectively inhibits type I IFN, shows promise in treating CLE. A recent case series demonstrated significant improvement in disease appearance, cutaneous involvement, and symptomatology after 2 months of anifrolumab infusions in CLE patients.^9^ A multicenter prospective study of 11 patients with systemic lupus erythematosus and active CLE found that all patients reported at least a 50% decrease in disease severity at week 16, highlighting anifrolumab's potential in refractory CLE.^10^

CLE/LP overlap syndrome can be debilitating and very frustrating to patients, especially when left undiagnosed for several years, as demonstrated in our case. Despite its devastating impact on quality of life, there are no established diagnostic criteria or consensus on treatment. Treatment is often based on the successful therapies for each separate disease entity. Previously reported treatments include systemic retinoids and cyclosporine.^4^ Here, we describe an interesting scenario where acitretin and anifrolumab were simultaneously started by different specialists for the treatment of CLE/LP overlap syndrome, leading to marked disease improvement. While it remains unclear whether both were necessary to achieve the degree of clearance observed, discontinuing acitretin has not been problematic thus far. The patient also experienced unexpectedly significant and rapid symptom relief. Future steps include monitoring progress on anifrolumab alone.

It is important to consider whether long-standing exposure to hydroxychloroquine caused drug-induced LP in this patient. This case of a patient with a prominent dermatologic history highlights the complexity of immunosuppressive therapy and emphasizes the importance of multidisciplinary care, maintaining strong patient rapport, and starting treatment interventions early to achieve good disease prognosis.

## Conflicts of interest

None disclosed.
